# Paradoxical pseudomyotonia in English Springer and Cocker Spaniels

**DOI:** 10.1111/jvim.15660

**Published:** 2019-11-14

**Authors:** Kimberley Stee, Mario Van Poucke, Luc Peelman, Mark Lowrie

**Affiliations:** ^1^ Dovecote Veterinary Hospital Derby United Kingdom; ^2^ Department of Nutrition, Genetics and Ethology Ghent University Merelbeke Belgium

**Keywords:** ATP2A1, Brody disease, Brody syndrome, canine, congenital pseudomyotonia, dog, exercise‐induced muscle stiffness, hypertonia, involuntary muscle contraction, myotonia, paramyotonia congenita, SCN4A

## Abstract

**Background:**

Paramyotonia congenita and Brody disease are well‐described conditions in humans, characterized by exercise‐induced myotonic‐like muscle stiffness. A syndrome similar to Brody disease has been reported in cattle. Reports of a similar syndrome in dogs are scarce.

**Objectives:**

To define and describe the clinical, diagnostic, and genetic features and disease course of paradoxical pseudomyotonia in Spaniel dogs.

**Animals:**

Seven client‐owned dogs (4 English Springer Spaniels and 3 English Cocker Spaniels) with clinically confirmed episodes of exercise‐induced generalized myotonic‐like muscle stiffness.

**Methods:**

Sequential case study.

**Results:**

All dogs were <24 months of age at onset. The episodes of myotonic‐like generalized muscle stiffness always occurred with exercise, and spontaneously resolved with rest in <45 seconds in all but 1 dog. Extreme outside temperatures seemed to considerably worsen episode frequency and severity in most dogs. Complete blood count, serum biochemistry including electrolytes, urinalysis, brain magnetic resonance imaging, cerebrospinal fluid analysis, electromyography, motor nerve conduction velocity, ECG, and echocardiography were unremarkable. Muscle biopsy samples showed moderate but nonspecific muscle atrophy. The episodes seemed to remain stable or decrease in severity and frequency in 6/7 dogs, and often could be decreased or prevented by avoiding the episode triggers. The underlying genetic cause is not identified yet, because no disease‐causing variants could be found in the coding sequence or splice sites of the 2 major candidate genes, *SCN4A* and *ATP2A1*.

**Conclusions and Clinical Importance:**

Paradoxical pseudomyotonia is a disease affecting Spaniels. It is of variable severity but benign in most cases.

**Abbreviations**
aa: amino acid
Ca^2+^: calcium
CK: creatine kinase
Cl^−^: chloride
CSF: cerebrospinal fluid
EMG: electromyography
K^+^: potassium
MNCV: motor nerve conduction velocity
MRI: magnetic resonance imaging
Na^+^: sodium

## 1 INTRODUCTION

Myotonia is defined as a delayed muscle relaxation after muscle activation, usually preceded by a period of rest, and improving with continuous activity (warm‐up phenomenon). In paradoxical myotonia, the delayed muscle relaxation occurs and worsens with repeated muscle activation (exercise), and improves with rest. On electromyography (EMG), distinctive myotonic discharges are identified in both myotonia and paradoxical myotonia.^1^ Pseudomyotonia is defined as delayed relaxation in muscle contraction after a voluntary movement, and is characterized by the absence of myotonic discharges on EMG.^2^

Paramyotonia congenita (OMIM #168300) is a skeletal muscle channelopathy of humans, characterized by nonpainful muscle stiffness exacerbated by cold (cold‐induced myotonia) and exertion (paradoxical myotonia), as well as possible episodes of weakness linked to the myotonic episodes (postmyotonic weakness). Altered membrane excitability is responsible for the characteristic delayed skeletal muscle relaxation as well as the episodes of weakness. During episodes of stiffness, muscles typically exhibit myotonic discharges on EMG. Causative variants of this disease so far are exclusively autosomal dominant and found in *SCN4A*, although in a subset of patients, no causal variant has been found.^3^, ^4^, ^5^, ^6^

Brody disease (OMIM #601003) is a skeletal myopathy, characterized by nonpainful muscle stiffness electrically silent on EMG (pseudomyotonia), occurring during exercise and worsened by cold exposure. Decreased sarcoplasmic reticulum Ca^2+^ ATPase (*SERCA1*) activity, causing decreased reuptake of Ca^2+^ by the sarcoplasmic reticulum at the end of muscle contraction, is responsible for delayed skeletal muscle relaxation.^7^, ^8^ Muscles typically are silent on EMG (pseudomyotonia) during an episode. This disease so far has been exclusively associated with homozygous or compound heterozygous recessive variants of *ATP2A1*.^9^, ^10^, ^11^ Brody syndrome is a phenotypically similar entity for which no causal variant has been found. It is unknown if Brody disease and Brody syndrome are the same entity or 2 different diseases resulting in a final common pathway.^11^ In cattle, a similar syndrome called congenital pseudomyotonia (OMIA #001464‐9913) has been reported and also is caused by homozygous or compound heterozygous recessive variants of *ATP2A1*.^2^, ^12^, ^13^

Clinically, paramyotonia congenita, Brody disease and Brody syndrome present in a similar way, with episodes of exercise‐induced generalized myotonic‐like muscle stiffness.^10^, ^11^ Diagnosis is made based on typical clinical signs, EMG testing during a myotonic‐like episode as well as genetic analysis.^4^, ^6^, ^7^, ^9^, ^10^ Reports of similar syndromes in dogs are scarce.^14^, ^15^

Here, we describe the clinical features of 7 dogs diagnosed with exercise‐induced generalized myotonic‐like episodes, as well as their diagnostic and genetic features.

## 2 MATERIALS AND METHODS

This case series was designed as a sequential hospital‐based case study with follow‐up. Dogs included were client‐owned dogs clinically diagnosed with episodes of exercise‐induced generalized myotonic‐like muscle stiffness. Inclusion criteria required video evidence of at least 1 typical episode of exercise‐induced muscle stiffness, assessment by a board‐certified neurologist, normalcy between episodes, and a questionnaire completed by the owner. Using history and phenotype, a diagnosis of paradoxical pseudomyotonia was suspected when a dog presented with myotonic‐like episodes triggered by strenuous exercise (eg, running, playing, and climbing stairs), which would sometimes evolve into stiff collapse in lateral recumbency. Clinical data were extracted, including signalment, age of onset of signs, duration and frequency of clinical signs, triggers, diagnostic tests performed, associated results, medications administered, disease course, and effect of the disease on quality of life.

General physical and neurological examinations were performed in all dogs, as well as CBC, serum biochemistry, including electrolytes (sodium [Na^+^], potassium [K^+^], chloride [Cl^−^], and calcium [Ca^2+^]) and creatine kinase (CK) activity. Pre‐ and post‐episode serum electrolyte (Na^+^, K^+^, Cl^−^, Ca^2+^) concentrations, CK activity, urinalysis, and urine fractional electrolyte excretion (Na^+^, K^+^, Cl^−^, and Ca^2+^) were determined in 3 dogs (Dogs 5, 6, and 7). Brain magnetic resonance imaging (MRI), cerebrospinal fluid (CSF) analysis, muscle biopsies of the pelvic and thoracic limb as well as echocardiography were performed in Dog 7. An EMG was performed in 3 dogs: conscious and during an episode in Dog 7, conscious and within minutes after an episode in Dog 5, and conscious as well as under sedation, at room temperature as well as after local application of ice and heat on the muscles of the lateral aspect of the thigh and shoulder in Dog 6. A motor nerve conduction velocity (MNCV) study also was performed in Dog 7, under general anesthesia. A commercially available electrophysiological unit (Medelec Synergy, Viasys Healthcare, Surrey, United Kingdom) was used for electrodiagnostic recordings. An ECG was performed in 2 dogs (Dogs 6 and 7). A commercially available ECG unit (Cardipia 200, Trismed co. LTD, Daejeon, South Korea) was used for ECG recordings. Diagnostic tests performed in each dog are included in the Supplementary Information files.

Because all known causal variants for paramyotonia congenita and Brody disease are single nucleotide variants or small insertions and deletions in the coding sequence or splice sites of *SCN4A* 4, 5, 6 and *ATP2A1*,^7^, ^10^, ^11^ respectively, a sequence analysis of the coding sequence and the splice sites of all coding exons of those genes was performed in Dog 5 in an attempt to identify the underlying genetic cause of paradoxical pseudomyotonia in Spaniel dogs. The predicted canine gene sequences were first compared to their reviewed human orthologs. Then, all coding exons were PCR amplified on genomic DNA extracted from an EDTA blood sample, Sanger sequenced and compared to the in silico validated canine reference sequence. Experimental details are described in the Supplementary Information files.

## 3 RESULTS

### 3.1 Patients

Eight client‐owned dogs, 5 English Springer Spaniels (3 males and 2 females, all neutered), and 3 Cocker Spaniels (2 neutered females and 1 intact male) were evaluated for episodes of exercise‐induced generalized myotonic‐like muscle stiffness. Seven of these dogs (4 English Springer Spaniels and the 3 Cocker Spaniels) were included in the study. A female sibling from 1 of the included English Springer Spaniels could not be included because no video footage of an episode was available.

### 3.2 Clinical features

All dogs were presented with their first episode between 3 and 24 months of age (median, 6 months). Episode frequency varied markedly among dogs, ranging from 15 episodes per day to 1 episode per year. The episodes only occurred during strenuous exercise and almost never indoors, with their frequency and severity being aggravated by stress (1 dog), excitement (4 dogs), and extremes of temperature (both hot [1 dog], cold [3 dogs], or both [2 dogs]), as well as by swimming in 3 dogs. A seasonal peak was observed during the summer (4 dogs), winter (3 dogs), or autumn (1 dog). A summary of these clinical features is available in the Supplementary Information files.

Episodes presented as a sudden onset of generalized myotonic‐like muscle stiffness while exercising, causing dogs to look like a dog “running in a computer game” (Video 1), get “stuck” while climbing stairs (Video 2), or while performing small jumps (Video 3). Episodes most commonly lasted for a few seconds and then spontaneously resolved with rest. Sometimes, the episode could present as rigid collapse in lateral recumbency (4/7 dogs, Video 4). This collapse would resolve spontaneously in <45 seconds in all but 1 (Dog 7) affected dogs. In Dog 7, episodes varied in duration, and severe episodes were associated with apnea and cyanosis.

### 3.3 Diagnostic tests and results

General physical and neurological examinations were unremarkable in all dogs. Five of the 7 dogs had moderate appendicular muscle hypertrophy. Complete blood count and serum biochemistry results were unremarkable in all dogs. In 2 dogs, pre‐ and post‐episode serum electrolytes concentrations (Na^+^, K^+^, Cl^−^, and Ca^2+^) were normal. Pre‐ and post‐episode CK activity was normal in 1 dog and mildly increased (pre‐episode, respectively: 303 and 367 U/L; post‐episode, respectively: 481 and 833 U/L; reference range, 20‐200 U/L) in 2 dogs. Urinalysis and fractional electrolyte excretion (Na^+^, K^+^, Cl^−^, and Ca^2+^) were normal in 3 dogs. Brain MRI, CSF analysis, echocardiography, and MNCV performed in 1 dog were normal. Electrocardiograms performed in 2 dogs were normal. Electromyography performed in 3 dogs (during, after, or in‐between episodes) failed to show the presence of any myotonic discharges. Muscle biopsies were performed in 1 dog and histology identified moderate but nonspecific myofiber atrophy in the pelvic limb muscles, and no abnormalities in the thoracic limb muscles.

### 3.4 Genetics

The predicted canine *SCN4A* gene is organized into 24 coding exons. Its longest isoform is encoded by all exons and is 1837 amino acids (aa) long. All 24 coding exons also are present in humans and the orthologous sequences show 90 and 94% sequence identity on coding sequence and aa level, respectively. Sequencing the coding sequence and the splice sites of all 24 coding exons of *SCN4A* of Dog 5 identified only 2 variants (already described) compared to the canine reference sequence, 1 silent, and 1 missense variant (NC_006591.3(XM_848303.4): c.299G>A (p.(Arg100Lys))). Because a Lys at position 100 is present in almost all mammalian reference sequences and is predicted to have a neutral effect (83%) on the protein by PredictSNP,^16^ the variant was not considered to be causal. During the analysis, the only gap in the canine *SCN4A* genome sequence in intron 22 (NC_006591.3:g.11855058_11855655inv) could be filled in (Acc. No. MN395478).

The predicted canine *ATP2A1* gene contains 23 coding exons. The 2 longest isoforms are encoded by 22 exons, both starting with the first 21 exons. Each ends with either exon 22 (994 aa long) or exon 23 (993 aa long). A similar organization is seen in humans and the orthologous sequences show 91 and 92% sequence identity, respectively, on coding sequence level and 96% on aa level. Sequencing the coding sequence and the splice sites of all 23 coding exons of *ATP2A1* of Dog 5 identified 5 (already described) silent variants compared to the canine reference sequence. Details on sequences and variants are given in the Supplementary Information files.

### 3.5 Disease course

Follow‐up period ranged from 6 months to 9 years (median, 4.5 years) and was assessed by telephone interview with the owners of the dogs, referring veterinarians or both. Episode frequency and severity generally remained stable or decreased over time in most dogs because episodes resolved in 2 dogs, only occurred when triggers like extreme hot or cold weather were present in 2 dogs, remained stable in 2 dogs, and worsened in 1 dog.

All but 1 owner rated their dog's quality of life as good to excellent, because these episodes most often could be decreased or eliminated by avoiding triggers. The remaining owner (Dog 7) estimated the dog's quality of life to be severely affected by the disease, because the dog could not even go for a normal walk without having a collapsing episode.

### 3.6 Treatment

Referring veterinarians had attempted to treat 4 dogs with various protocols before referral, including phenobarbital (Dogs 1, 3, and 4); nonsteroidal anti‐inflammatory drugs and various antibiotics (Dog 3); diazepam, butylhyoscine bromide, and methocarbamol (Dog 4); and mexiletine and levetiracetam (Dog 7); no or very mild improvement had been noticed using those drugs. Given the apparent benign nature of this condition in most dogs, the fact that episodes did not progress and the fact that they often could be decreased or eliminated by avoiding triggers, medical treatment did not seem necessary for 6/7 dogs. Given the severity of the episodes in Dog 7, an attempted treatment with clonazepam (0.5 mg/kg PO q8h) was initiated. Because the adverse effects were too severe (unable to walk because of marked sedation and ataxia), the dosage was gradually decreased to 0.125 mg/kg PO q8h, which markedly decreased the severity and frequency of the paradoxical pseudomyotonia episodes with acceptable adverse effects (polyphagia, ptyalism, and mild sedation).

## 4 DISCUSSION

We summarized the clinical, diagnostic and genetic features, as well as disease course, of 7 dogs with episodes of exercise‐induced generalized myotonic‐like muscle stiffness. The phenotype of our dogs displays striking similarities with the paramyotonia congenita and Brody disease phenotypes in people, as well as with congenital pseudomyotonia in cattle, because they all present as episodes of exercise‐induced generalized myotonic‐like muscle stiffness that seems to be aggravated by cold. Differences include the worsening of the myotonic‐like episodes by hot weather in some of our dogs, and the absence of episodic weakness in all of our dogs. Diagnosis in humans is based on typical clinical features, the presence or absence of myotonic bursts on EMG as well as genetic testing.^4^, ^6^, ^7^, ^9^, ^10^ Because no myotonic discharges were present on EMG during an episode and no causal variant was found in the coding sequence or splice sites of *SCN4A* or *ATP2A1*, we conclude that our dogs seem to be affected with the canine counterpart of Brody syndrome, and propose to refer to this syndrome as paradoxical pseudomyotonia.

Human patients with Brody disease or syndrome and cattle with congenital pseudomyotonia do not suffer from respiratory signs.^2^, ^7^, ^10^, ^11^, ^12^, ^13^ Newborn *SERCA1* null mice die of respiratory failure within 2 hours of birth because of severely impaired diaphragmatic function. Because *SERCA1* is only found in fast twitching (Type II) fibers,^17^ this phenotypical difference might be explained by the different percentages of fast‐twitch and slow‐twitch muscle fibers in the diaphragm of those species, respectively, 50:50 for humans, 24:76 for cattle, and 93:7 for mice.^18^, ^19^ One of our dogs was reported to experience apnea and cyanosis during severe episodes, which could be a consequence of diaphragmatic muscle stiffness. The diaphragmatic fast‐twitch: slow‐twitch muscle fiber ratio in dogs is 64:36,^18^ which would be in accordance with the hypothesis that the more fast twitch fibers present, the more prone a species or individual might be to diaphragmatic stiffness during an episode of generalized pseudomyotonia.

Local application of ice on muscles while performing the EMG has been described in some human patients as being the only way to unmask myotonic bursts.^1^ This was attempted in 1 of our dogs. Heat application on the muscles also was performed, because some of our dogs also showed worsening of their muscle stiffness with extremely hot temperatures. None of those applications unmasked any myotonic discharges.

Treatment of Brody disease or syndrome currently is unsatisfactory. Clonazepam currently is the drug with the highest treatment success rate.^11^ It has muscle relaxant and anxiolytic properties^20^ and has been described to have moderate positive effect, and no prominent adverse effects. Dantrolene and verapamil have been used in human patients with very limited positive effect and marked adverse effects including muscle weakness, depression, and malaise.^11^ Dantrolene has the most specific mode of action as it is believed to exert its muscle relaxant effect by interacting with Ca^2+^ release from the sarcoplasmic reticulum, but this drug has been associated with hepatotoxicity with long‐term use.^20^ Given its harmless nature and highest success rate for producing a positive effect on pseudomyotonia episodes in humans, clonazepam was considered the first treatment of choice to treat Dog 7.

Given the close historical relationship between the English Springer Spaniel and the English Cocker Spaniel breeds,^21^, ^22^ it was speculated that the same genetic cause could be responsible for this disorder in both breeds. No causal variant was found in the coding sequence and the splice sites of the *SCN4A* or *ATP2A1* genes. Similarly, in 29% of human patients with a clinical phenotype typical for paramyotonia congenita^6^ as well as in patients with Brody syndrome,^11^ no causal variants were found, suggesting further genetic heterogeneity.^4^, ^6^, ^11^ Limitations of our study include its partially retrospective nature, the small number of dogs included, the lack of a diagnostic test confirming the diagnosis, and a genetic analysis specifically aiming for variants similar to those that were already described.

In conclusion, paradoxical pseudomyotonia seems to be a disorder segregating in the English Springer and Cocker Spaniel breeds. This disease is of variable severity, but seems to be a benign, nonpainful and nonprogressive disorder in most cases. Pseudomyotonia episodes often can be decreased or eliminated by avoiding episode triggers, such that treatment often does not seem necessary. Quality of life of dogs affected with this disease is estimated to be good to excellent for most dogs. In some cases, the disease can be much more prominent, worsen with time, impair the patient's quality of life and cause episodes of apnea paired with severe pseudomyotonia episodes. If necessary, treatment with clonazepam can be attempted. The genetic variant responsible for this disorder currently remains unknown, and further genetic studies will be required to discover the causal variant.

## CONFLICT OF INTEREST DECLARATION

Authors declare no conflict of interest.

## OFF‐LABEL ANTIMICROBIAL DECLARATION

Authors declare no off‐label use of antimicrobials.

## INSTITUTIONAL ANIMAL CARE AND USE COMMITTEE (IACUC) OR OTHER APPROVAL DECLARATION

Authors declare no IACUC or other approval was needed.

## HUMAN ETHICS APPROVAL DECLARATION

Authors declare human ethics approval was not needed for this study.

## Supporting information


**Supplementary information file 1**: Details on individual clinical data.

**Supplementary information file 2**: Details on the genetic analysis.

**Video 1** Dog 5 displaying an episode of paradoxical pseudomyotonia while running.

**Video 2** Dog 1 displaying an episode of paradoxical pseudomyotonia while climbing stairs.

**Video 3** Dog 5 displaying an episode of paradoxical pseudomyotonia while performing a small jump.

**Video 4** Dog 6 displaying an episode of paradoxical pseudomyotonia causing him to fall in a rigid collapse.
