# Supplementary material for: Paradoxical pseudomyotonia in English Springer and Cocker Spaniels
Source: J Vet Intern Med. 2019 Nov 14;34(1):253–7. doi: 10.1111/jvim.15660 (PMC6979413; doi:10.1111/jvim.15660)
Supplement: Supplementary file 1 — Supplementary information file 1: Details on individual clinical data. [file JVIM-34-253-s001.pdf]

Supplementary Information File. Details on individual clinical data

1. **Diagnostic tests performed in each of the 7 dogs of the study**

Dog	1	2	3	4	5	6	7
Blood work	+	+	+	+	+	+	+
Urinalysis and excretion fraction	-	-	-	-	+	+	+
Pre and post exercise electrolytes and CK	-	-	-	-	+	+	+
EMG	-	-	-	-	+	+	+
MNCV	-	-	-	-	-	-	+
ECG	-	-	-	-	-	+	+
Echocardiography	-	-	-	-	-	-	+
MRI brain and CSF	-	-	-	-	-	-	+
Muscle biopsies	-	-	-	-	-	-	+

CK: creatine kinase, EMG: electromyography, MNCV: Motor nerve conduction velocity, MRI: magnetic resonance imaging, CSF: cerebrospinal fluid, +: performed, -: not performed.

2. Summary of the clinical features of the phenotype of the 7 dogs of the study

Dog	1	2	3	4	5	6	7
Breed	ESS	ECS	ECS	ESS	ESS	ECS	ESS
Sex	MN	FN	FN	FN	MN	ME	MN
Age (y)	11	6	9	5	1,5	1	7
Onset (m)	24	18	6	16	3	3	3
Frequency	1-2/y	2-3/w	5 (life)	1/m	2-3/m	2-15/day	2/d
Triggers							
- exercise	+	+	+	+	+	+	+
- excitement	+	-	-	+	+	+	+
- cold weather	+	+	+	-	+	+	-
- hot weather	+	-	-	+	+	-	-
- swimming	+	-	+	-	-	-	+
- stress	-	-	-	-	-	-	+
Evolution	Improved	Resolved	Resolved	Improved	Stable	Stable	Worsened
Seasonal peak							
- Summer	+	-	-	+	+	-	+
- Winter	+	-	-	-	+	NA	-
- Autumn	-	-	-	-	-	NA	+

ESS: English Springer Spaniel, ECS: English Cocker Spaniel, MN: male neutered, FN: female neutered, ME: male entire, y: year, m: month, w: week, d: day, life: over whole life, NA: non-applicable.
