# Supplementary material for: Paradoxical pseudomyotonia in English Springer and Cocker Spaniels
Source: J Vet Intern Med. 2019 Nov 14;34(1):253–7. doi: 10.1111/jvim.15660 (PMC6979413; doi:10.1111/jvim.15660)
Supplement: Supplementary file 2 — Supplementary information file 2: Details on the genetic analysis. [file JVIM-34-253-s002.pdf]

Supplementary Information File. Details on the genetic analysis.

1. Overview
2. Gene sequences
3. Canine exon description
4. Primer sequences
5. PCR assays
6. PCR mixes
7. PCR program
8. EXO/AP-mix
9. EXO/AP-treatment
10. Sequencing mix
11. Sequencing program
12. Canine SCN4A intron 22 sequence
13. Variants
14. References

1. Overview

A sequence analysis of the coding sequence and splice sites of all coding exons of SCN4A and ATP2A1 was performed on genomic DNA of affected dog 5 (Table 1). DNA was isolated from EDTA blood by performing a proteinase K digestion. The predicted canine gene sequences were first compared to their reviewed human orthologs. The SCN4A transcript variant contains all known coding exons. The ATP2A1 transcript variants are identical except for a different last exon (exon 22 or 23) and comprise all known coding exons. Primers amplifying all coding exons were designed with Primer-Blast (Ye *et al*, 2012) based on the canine genomic reference sequence (CanFam 3.1). PCR amplicons were EXO/AP-treated, Sanger sequenced and sequences were compared with the canine genomic reference sequence. During sequencing, the gap in the canine SCN4A intron 20 reference sequence (NC_006591.3:g.11855058_11855655inv) was resolved.

2. Gene sequences (NCBI)

Sequence	Human ID (length)	Canine ID (length)	Sequence ID (human/canine)
SCN4A Gene	6329	610754	na
SCN4A Gene Sequence	NG_011699.1	NC_006591.3	na
SCN4A CDS 1	NM_000334.4 (5511 bp)	XM_848303.4 (5514 bp)	90 %
SCN4A Isoform 1	NP_000325.4 (1836 aa)	XP_853396.1 (1837 aa)	94 %
ATP2A1 Gene	487	479797	na
ATP2A1 Gene Sequence	NG_023327.1	NC_006588.3	na
ATP2A1 CDS 1	NM_004320.5 (2985 bp)	XM_855027.5 (2982 bp)	91 %
ATP2A1 Isoform 1	NP_004311.1 (994 aa)	XP_860120.1 (993 aa)	96 %
ATP2A1 CDS 2	NM_173201.4 (3006 bp)	XM_854917.5 (3003 bp)	92 %
ATP2A1 Isoform 2	NP_775293.1 (1001 aa)	XP_860010.2 (1000 aa)	96 %

3. Canine exon description

Exon (CDS)	SCN4A (NC_006591.3)	ATP2A1 (NC_006588.3)
1	complement(11877495..11877767)	18385185..18385302
2	complement(11877287..11877405)	18385630..18385647
3	complement(11877066..11877155)	18386064..18386146
4	complement(11876667..11876795)	18387495..18387599
5	complement(11876087..11876178)	18388250..18388388
6	complement(11874196..11874510)	18390320..18390400
7	complement(11873312..11873375)	18391253..18391338

8	complement(11872936..11873077)	18391494..18391791
9	complement(11871969..11872178)	18391960..18392126
10	complement(11871231..11871384)	18393875..18393963
11	complement(11869344..11869582)	18394238..18394340
12	complement(11867396..11867569)	18394544..18394675
13	complement(11866079..11866435)	18395784..18395906
14	complement(11861567..11862064)	18396035..18396253
15	complement(11859537..11859669)	18396970..18397305
16	complement(11858747..11858901)	18397465..18397685
17	complement(11858056..11858229)	18397799..18398001
18	complement(11857810..11857932)	18398188..18398273
19	complement(11855969..11856247)	18398366..18398499
20	complement(11855656..11855709)	18398624..18398741
21	complement(11854920..11855057)	18398906..18399023
22	complement(11854333..11854437)	18399273..18399277
23	complement(11853343..11853613)	18399753..18399778
24	complement(11851347..11852572)	

4. Primer sequences (IDT)

Primer name	Gene location	Primer sequence (5’ -> 3’)
SCN4A-1F	SCN4A-EX1 (5’-UTR)	ACCAAGGCAGGGAGGAGGTGAGTA
SCN4A-1R	SCN4A-IN2	GGGAGAGTCAAGGAAAGTAAGGGAGCA
SCN4A-2F	SCN4A-EX2	GCTTCTCTGCCACACCCGCT
SCN4A-2R	SCN4A-IN4	CCCACTAGCAGTCCACCTCCCA
SCN4A-3F	SCN4A-IN4	GCCTAGAGAAGCTGTGATTTCTGCCC
SCN4A-3R	SCN4A-IN5	TGTGGTGTGAGAGTGTTGTGACCC
SCN4A-4F	SCN4A-IN5	AGGTGGGACTCATTTGGGAAGCC
SCN4A-4R	SCN4A-IN6	GGGTTCTGAGGGTTAGGGAGACCA
SCN4A-5F	SCN4A-IN6	CGTGCGATGGGTGCATGTGT
SCN4A-5R	SCN4A-IN8	AGTGGCCTTGGTCCTGCCAT
SCN4A-6F	SCN4A-IN8	GCGACCCTCCCTGTTTACCTATCTG
SCN4A-6R	SCN4A-IN9	TTCCCATCCCACCAAAACCTCCA
SCN4A-7F	SCN4A-IN9	TGGTCCCTACCCTGGTCAATCTCA
SCN4A-7R	SCN4A-IN10	GCCCAGAAGACATTTGAGGAGCAG
SCN4A-8F	SCN4A-IN10	GGGAGCATGAGTGGGCAGGGTTT
SCN4A-8R	SCN4A-IN11	GCCCAGCTTGTCCCCATATGCCC
SCN4A-9F	SCN4A-IN11	CCCAGAAGCAGATGGGTTCAGGT
SCN4A-9R	SCN4A-IN12	GTCAAGCCCAAATGTCAGCTCCC
SCN4A-10F	SCN4A-IN12	GGCTCAGATCACTCGTGAGGCA
SCN4A-10R	SCN4A-IN13	TGGTTACAATTGGAGAGTCACTGGGT
SCN4A-11F	SCN4A-IN13	GTCTTGCAGAGTCCTATCGTGCG

SCN4A-11R	SCN4A-IN14	GAGGTGCAGAGCCAAAGTGAGG
SCN4A-12F	SCN4A-IN14	TGAATGACTGAGTAGGTCAGCTCCAC
SCN4A-12R	SCN4A-IN15	CGGTGCTATACCCACGGCCA
SCN4A-13F	SCN4A-IN15	ACCAAGCCAGCCCTTCAGACAC
SCN4A-13R	SCN4A-IN16	CGCATCCTGCAACCACAAATGAGT
SCN4A-14F	SCN4A-IN16	AGAAGCCAATAGCCTGACCCCA
SCN4A-14R	SCN4A-IN18	CTTTAGCAGGACCCAGAGACCGA
SCN4A-15F	SCN4A-IN18	ATAACAGTGCCCAGCCCCGATG
SCN4A-15R	SCN4A-IN20	TGTCATGTGCTTCTGATCTGCGTCC
SCN4A-16F	SCN4A-IN19	TGGTGGAAATGAGAAGCAGCCCC
SCN4A-16R	SCN4A-IN21	GCTGAGGTGGCAGGTGGGAATA
SCN4A-17F	SCN4A-IN21	AGGGTGGTGCCTGAGGTAGGG
SCN4A-17R	SCN4A-IN22	CCTCACGCCAGGGATATGGGC
SCN4A-18F	SCN4A-IN22	TCATCTTGTGTCTCCTGCCTGTCC
SCN4A-18R	SCN4A-IN23	TGTATGTGTGAGCTTTCCAGGTGTG
SCN4A-19F	SCN4A-IN23	CCTCACGGGCCAGGTCATTTAGA
SCN4A-19R	SCN4A-EX24	TGAGAGGCGGCTGTAGGCAAT
SCN4A-20F	SCN4A-EX24	GGCCACCGAGGAGAGCAGTG
SCN4A-20R	SCN4A-EX24 (3'-UTR)	GCCGATCAGTTGCAGACTCAAAGC
SCN4A-21R	SCN4A-EX18	TCAAATCGGGACAGAGCCCTCA
ATP2A1-1F	ATP2A1-EX1 (5'-UTR)	AAAGCAAGAAACCCAGGCAAACAGG
ATP2A1-1R	ATP2A1-IN2	ATTTGCCCCGGGAGTCAGTAGG
ATP2A1-2F	ATP2A1-IN2	CCAGTTTGTGGAGCCCGTTTCA
ATP2A1-2R	ATP2A1-IN3	GATGGTGAGGACTTGGACCCGA
ATP2A1-3F	ATP2A1-IN3	TGCACACACACTCACCCTCACTATG
ATP2A1-3R	ATP2A1-IN4	ATCTCTTTCCTGCACTCCATCCCCT
ATP2A1-4F	ATP2A1-IN4	TCCTCAACACAGACACCTACCCTCA
ATP2A1-4R	ATP2A1-IN5	GCAAAGACTCCACCATCCCAGCTA
ATP2A1-5F	ATP2A1-IN5	CTGAGGACGAGGTGAGGTTGTCAG
ATP2A1-5R	ATP2A1-IN6	CCCCTTTTGTTTCAGGCTTGCCAT
ATP2A1-6F	ATP2A1-IN6	TCCAGTGTTCAGCCTTCCTCCAG
ATP2A1-6R	ATP2A1-IN8	GGAACTTGAAAATGTTACCCTGCTTGC
ATP2A1-7F	ATP2A1-IN8	GCTAATCTGAACTGCAAAGGCCCC
ATP2A1-7R	ATP2A1-IN9	TGGCAGGGTGACAATCTATACCTGT
ATP2A1-8F	ATP2A1-IN9	TCTACAAGGGTGGTTGGGAGAGGAA
ATP2A1-8R	ATP2A1-IN10	AAGAGTGAGGGACAGTCAGGGACG
ATP2A1-9F	ATP2A1-IN10	TCTCCATGCTGGTTGCCACGTC
ATP2A1-9R	ATP2A1-IN12	TCCCCTGAGGTTCTCTGGTCCTTG
ATP2A1-10F	ATP2A1-IN12	GCCCTTCTAGCACAGCATCATCATT
ATP2A1-10R	ATP2A1-IN14	CAGCTACCACCTGGGTCTCTGT
ATP2A1-11F	ATP2A1-IN14	GAGCTTCCTCCCTGGCTTACTTCCA

ATP2A1-11R	ATP2A1-EX17/IN16	AAGATGCTGCCAGAAGGAGGTGGT
ATP2A1-12F	ATP2A1-EX16	GCAGTTCATCCGCTACCTCATCTCC
ATP2A1-12R	ATP2A1-EX19	TCGCAGTCCACGCCCTCAAAA
ATP2A1-13F	ATP2A1-EX18	TATGCAGATGACGGGCCTGGTGT
ATP2A1-13R	ATP2A1-IN20	TGGAGGTGGTGAGGACTGGTGTTC
ATP2A1-14F	ATP2A1-IN20	ACGCAGACAGAGGGAGGAAAGGGTG
ATP2A1-14R	ATP2A1-IN22	TGGGAAGCGTGGCAGTGGCAG
ATP2A1-15F	ATP2A1-IN22	ACAGAGAGCCTGAGAGGGACGTT
ATP2A1-15R	ATP2A1-EX23 (3'-UTR)	TGAGGGTGCAAAGTGGAAGGGAC

5. PCR assays

**Exon**	**PCR primers**	**PCR mix (Ta)**	**Amplicon length (bp)**	**Sequencing primer**
SCN4A-1	SCN4A-1F-1R	1 (65°C)	739	SCN4A-1F
SCN4A-2	SCN4A-1F-1R	1 (65°C)	739	SCN4A-1R
SCN4A-3	SCN4A-2F-2R	1 (65°C)	811	SCN4A-2F
SCN4A-4	SCN4A-2F-2R	1 (65°C)	811	SCN4A-2R
SCN4A-5	SCN4A-3F-3R	1 (65°C)	304	SCN4A-3F
SCN4A-6	SCN4A-4F-4R	1 (65°C)	592	SCN4A-4F
SCN4A-7	SCN4A-5F-5R	2 (64°C)	604	SCN4A-5F
SCN4A-8	SCN4A-5F-5R	2 (64°C)	604	SCN4A-5R
SCN4A-9	SCN4A-6F-6R	2 (64°C)	479	SCN4A-6F
SCN4A-10	SCN4A-7F-7R	2 (64°C)	650	SCN4A-7F
SCN4A-11	SCN4A-8F-8R	1 (67°C)	581	SCN4A-8R
SCN4A-12	SCN4A-9F-9R	2 (64°C)	640	SCN4A-9R
SCN4A-13	SCN4A-10F-10R	2 (64°C)	696	SCN4A-10F
SCN4A-14	SCN4A-11F-11R	1 (63°C)	1009	SCN4A-11R
SCN4A-15	SCN4A-12F-12R	2 (64°C)	613	SCN4A-12F
SCN4A-16	SCN4A-13F-13R	2 (64°C)	501	SCN4A-13F
SCN4A-17	SCN4A-14F-14R	1 (63°C)	670	SCN4A-21R
SCN4A-18	SCN4A-14F-14R	1 (63°C)	670	SCN4A-14R
SCN4A-19	SCN4A-15F-15R	1 (65°C)	773	SCN4A-15F
SCN4A-20	SCN4A-15F-15R	1 (65°C)	773	SCN4A-15R
SCN4A-21	SCN4A-16F-16R	2 (64°C)	793	SCN4A-16R
SCN4A-22	SCN4A-17F-17R	1 (65°C)	299	SCN4A-17R
SCN4A-23	SCN4A-18F-18R	2 (64°C)	470	SCN4A-18F
SCN4A-24 (5')	SCN4A-19F-19R	2 (64°C)	788	SCN4A-19F
SCN4A-24 (3')	SCN4A-20F-20R	2 (64°C)	808	SCN4A-20F
ATP2A1-1	ATP2A1-1F-1R	1 (64°C)	681	ATP2A1-1F
ATP2A1-2	ATP2A1-1F-1R	1 (64°C)	681	ATP2A1-1R
ATP2A1-3	ATP2A1-2F-2R	1 (64°C)	388	ATP2A1-2F
ATP2A1-4	ATP2A1-3F-3R	1 (65°C)	305	ATP2A1-3F

ATP2A1-5	ATP2A1-4F-4R	1 (64°C)	475	ATP2A1-4F
ATP2A1-6	ATP2A1-5F-5R	1 (64°C)	377	ATP2A1-5R
ATP2A1-7	ATP2A1-6F-6R	1 (64°C)	762	ATP2A1-6F
ATP2A1-8	ATP2A1-6F-6R	1 (64°C)	762	ATP2A1-6R
ATP2A1-9	ATP2A1-7F-7R	1 (64°C)	459	ATP2A1-7F
ATP2A1-10	ATP2A1-8F-8R	1 (65°C)	391	ATP2A1-8R
ATP2A1-11	ATP2A1-9F-9R	1 (65°C)	641	ATP2A1-9F
ATP2A1-12	ATP2A1-9F-9R	1 (65°C)	641	ATP2A1-9R
ATP2A1-13	ATP2A1-10F-10R	1 (63°C)	776	ATP2A1-10F
ATP2A1-14	ATP2A1-10F-10R	1 (63°C)	776	ATP2A1-10R
ATP2A1-15	ATP2A1-11F-11R	1 (65°C)	900	ATP2A1-11F
ATP2A1-16	ATP2A1-11F-11R	1 (65°C)	900	ATP2A1-11R
ATP2A1-17	ATP2A1-12F-12R	1 (65°C)	784	ATP2A1-12F
ATP2A1-18	ATP2A1-12F-12R	1 (65°C)	784	ATP2A1-12R
ATP2A1-19	ATP2A1-13F-13R	1 (65°C)	658	ATP2A1-13R
ATP2A1-20	ATP2A1-13F-13R	1 (65°C)	658	ATP2A1-13R
ATP2A1-21	ATP2A1-14F-14R	1 (67°C)	658	ATP2A1-14F
ATP2A1-22	ATP2A1-14F-14R	1 (67°C)	658	ATP2A1-14R
ATP2A1-23	ATP2A1-15F-15R	1 (64°C)	241	ATP2A1-15F

6. PCR mixes (VWR International)

PCR mix 1
5.7 µl H_2O
1.0 µl 10x Key buffer
0.0 µl GC-rich (Roche)
1.0 µl Primer mix (5 µM each primer)
0.2 µl dNTPs (10 mM each nucleotide)
0.1 µl TEMPase HS DNA Polymerase (5 U/µl)
2.0 µl DNA (10 ng/µl)
10.0 µl Total volume

PCR mix 2
4.7 µl H_2O
1.0 µl 10x Key buffer
1.0 µl GC-rich (Roche)
1.0 µl Primer mix (5 µM each primer)
0.2 µl dNTPs (10 mM each nucleotide)
0.1 µl TEMPase HS DNA Polymerase (5 U/µl)
2.0 µl DNA (10 ng/µl)
10.0 µl Total volume

7. PCR program (S1000 thermal cycler, Bio-Rad Laboratories)

14’30” - 95°C
00’30” - 95°C]
00’30” - Ta°C] x35
01’00” - 72°C]
02’00” - 72°C
HOLD – 15°C

8. EXO/AP-mix

10,0 µl PCR product
0,6 µl EXO/AP (4 U exonuclease I + 2 U antarctic phosphatase, Biolabs)

9. EXO/AP-treatment

30’ - 37°C
15’ - 80°C

10. Sequencing Mix (BigDye Terminator v3.1 Cycle Sequencing Kit, Applied Biosystems)

3.0 µl H_2O
0.5 µl RR-mix
2.0 µl 5x sequencing buffer
1.0 µl GC-rich (Roche)
1.5 µl sequencing primer (2 µM)
2.0 µl EXO/AP-treated PCR product
10.0 µl total volume

11. Sequencing program

02’00” - 95°C
00’20” - 95°C]
00’15” - 60°C] x30
04’00” - 65°C]
HOLD - 04°C

Sequencing reactions were run on an ABI 3730 XL sequencing machine at Eurofins Genomics (Germany) and sequence analysis was performed with Unipro UGENE v1.16.1 (Okonechnikov *et al*, 2012)

12. Canine SCN4A intron 22 sequence (Acc. No. MN395478)

```
GTGAGTGGGG CTCACTGAGA TGTGGCTGGC AAGTGAGCCA GGGGAGAGGA CGCAGATCAG    60
AGGCACAATG ACAGTTCTGC CCAACCCCAC CCCCATTCCA CCTCCTCAGG AGCCCCCAAA   120
ATCAGCAACC TGGGGGTGGA AGGATCCACC TCTGGGTCCC AGACTCCTCC TGCCCACAGT   180
GAGATGGGGA GCCGGGAGCA GCCAACATAC TCCCCCCAAC CTCCCTATCC TGTCCCATCC   240
CATCCTGTTC CATCCCCATC CTATCCCATC CCATCCTCTC CCATCCCATC CCATCCTGTC   300
CCATCCCTTC CCCTCCTATC TCATCCCATC CCAGCCCCAT CCCATCCCAT CCTCATTCTA   360
TCTTGTTCCA TCCTGTCCCA TCCCCATCCC CATCCCCATC CCATCCCATC CCATCGCATC   420
CCATCACATC CCATCCCCAT CCCATCTTGG AGCCCCATCC CATCCTGCCC TGTGTCCTCT   480
GCAG                                                                484
```

13. Variants

Gene - Exon	Variant description	Type	Genotype	Variant ID
SCN4A - Ex2	NC_006591.3(XM_848303.4):c.299G>A (p.(Arg100Lys))	Missense	Vt/Vt	rs852470803
SCN4A - Ex24	NC_006591.3(XM_848303.4):c.4644C>T (p.(Ser1548Ser))	Silent	Vt/Vt	rs24600437
ATP2A1 - Ex6	NC_006588.3(XM_854917.5):c.513G>A (p.(Gln177Gln))	Silent	Vt/Vt	rs24261507
ATP2A1 - Ex8	NC_006588.3(XM_854917.5):c.813T>C (p.(Val271Val))	Silent	Vt/Vt	rs24337120
ATP2A1 - Ex15	NC_006588.3(XM_854917.5):c.1965A>G (p.(Arg655Arg))	Silent	Vt/Vt	rs850890561
ATP2A1 - Ex15	NC_006588.3(XM_854917.5):c.1998T>G (p.(Arg666Arg))	Silent	Vt/Vt	rs851932800
ATP2A1 - Ex19	NC_006588.3(XM_854917.5):c.2718A>C (p.(Ile906Ile))	Silent	Vt/Vt	rs24307979

14. References

Okonechnikov K, Golosova O, Fursov M, the UGENE team. Unipro UGENE: a unified bioinformatics toolkit. *Bioinformatics* 2012; **28:** 1166-1167.

Ye J, Coulouris G, Zaretskaya I, Cutcutache I, Rozen S, Madden T. Primer-BLAST: A tool to design target-specific primers for polymerase chain reaction. *BMC Bioinformatics* 2012; **13**: 134.
